# A comparative study to assess the production of two oilseed crops (*Brassica carinata* A. Braun and *Carthamus tinctorius* L.) and the energy potential of their agricultural biomass residues

**DOI:** 10.1016/j.heliyon.2024.e38654

**Published:** 2024-09-28

**Authors:** Mario Licata, Davide Farruggia, Filippo Sgroi, Francesco Salamone, Claudio Leto, Giuseppe Di Miceli

**Affiliations:** a Department of Agricultural, Food and Forest Sciences, Università degli Studi di Palermo, Viale delle Scienze 13, Building 4, 90128, Palermo, Italy; b Research Consortium for the Development of Innovative Agro-Environmental Systems, Via della Libertà 203, 90143, Palermo, Italy

**Keywords:** Agricultural residue, Biomass potential, Energetic properties, Ethiopian mustard, Safflower

## Abstract

Ethiopian mustard (*Brassica carinata* A. Braun) and safflower (*Carthamus tinctorius* L.) are two oilseed crops which have been largely developed as biofuel feedstock. Their agricultural residues can be also valorised for generation of renewable energy directly on-farm conforming to sustainability goals. This study aimed to compare the yield performances of Ethiopian mustard and safflower under rainfed conditions, assess the energetic characteristics of agricultural residue and pellet and calculate the agricultural residue energy potential.

Two-year tests were carried out in Sicily (Italy) comparing 20 accessions of Ethiopian mustard and 19 accessions of safflower. Seed yield (SY) and agricultural residue yield (ARY) were obtained on a harvest area of 7 m^2^. Ash content, moisture content, higher heating value (HHV) and lower heating value (LHV) of the agricultural residues were calculated. HHV and mechanical durability of pellets were recorded. Theoretical biomass potential (TBP), theoretical biomass energy potential (TEP) and available biomass energy potential (AEP) of residues were subsequently determined. The economic impact derived by cultivation of the two crops was also evaluated. Ethiopian mustard produced the highest average SY (2.9 t ha^−1^) and ARY (7.8 t ha^−1^), in both growing seasons. Significant differences were found concerning agricultural residues in moisture content and non-significant differences in HHV and LHV in both species. The pellets made from the two oilseed crops residues were modestly suited for energy use. Ethiopian mustard performed better than safflower in terms of TEP (91.27 GJ ha^−1^) and AEP (66.63 GJ ha^−1^) and could be considered of greater interest for valorisation of agricultural residues to renewable energy. These findings confirm the good productivity of the two crops in the Southern Mediterraean area and, particularly, highlight the greatest economic profitability of safflower (484 € ha^−1^) when considering also the seed sale price.

## Highlights

• Ethiopian mustard and safflower show relevant differences in agricultural residue yields.
• Agricultural residue is a valuable feedstock for producing bioenergy directly on-farm.
• The pellet made from agricultural residue is modestly suited to energy use.
• Biomass potential of crops allows to plan energetic production based on residues.
• Ethiopian mustard is the most convenient for energy purpose based on residues.

## Graphical abstract

**Figure:**
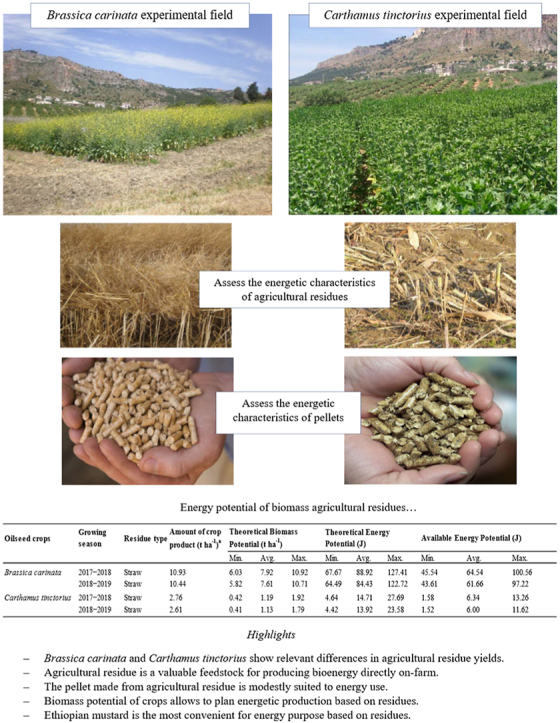

**Table:** Nomenclature

AEP	Available biomass energy potential	joule per hectare (J ha^−1^)
ARY	Agricultural residue yield	tonne per hectare (t ha^−1^)
HHV	Higher heating value	megajoule per kilogram (MJ kg^−1^)
LHV	Lower heating value	megajoule per kilogram (MJ kg^−1^)
MD	Mechanical durability	percentage value (%)
SY	Seed yield	tonne per hectare (t ha^−1^)
TBP	Theoretical biomass potential	tonne per hectare (t ha^−1^)
TEP	Theoretical biomass energy potential	gigajoule per hectare (GJ ha^−1^)
TSW	Thousand seed weight	gram (g)
TBY	Total biomass yield	tonne per hectare (t ha^−1^)

## 1 Introduction

Over the last twenty years, energy consumption and growing environmental consciousness have led to an increasing interest in renewable energy sources. The impact of conventional fossil fuels on global warming and the effect of greenhouse gas (GHG) emissions on the environment have negatively affected human health and economic growth across the world, as stated by several studies [1,2]. In an effort to decarbonize the energy sector and promote climate change mitigation, the contribution of renewable energy has become increasingly more prominent, thus reducing the stock of fossil fuels in the energy mix [3]. The reduction of GHG emissions and climate change mitigation through the use of renewable energies is largely encouraged by the European Commission, which has suggested a target of net zero GHG emissions to realized a climate neutral EU by 2050, in accordance with other international agreements on climate [4,5].

Renewable sources include wind, solar, hydroelectric, geothermal and biomass energies and represent the most important part of the energy transition based on sustainable criteria for the exploitation of environmental resources [6]. Recent studies show that plant biomass can significantly contribute to the green transition by removing carbon dioxide from the atmosphere, storing it for varying periods of time, and then exploiting it by directly replacing fossil fuel [[7], [8], [9]]. Some authors affirm that energy from biomass can also address the inconstancy of solar and wind energies, and play a crucial role in the circular economy as one of the main goals of the European Green Deal [10]. Within the European Union in particular, wood biomass represents the most exploited renewable energy source of all the various types of biomass; however, the use of trees for energy production is expected to be minimal in the next future, because of the EU Biodiversity Strategy for 2030 [11,12]. Undoubtedly, in line with the Directive (EU) 2018/2001 of the European Parliament and of the Council [13], use of biomass for energy purposes must ensure that there are no negative consequences for the environment in terms of deforestation or loss of plant biodiversity, and decisions on its use should be made considering the principles of recycling, reuse and disposal of agricultural and forestry residues, as well reported by Gupta et al. [14]. This concept was previously expressed by Scarlat et al. [15], who affirmed that the exploitation of biomass must include all available resources in a sustainable manner reducing the negative impacts. Furthermore, as highlighted in some studies, there should not be direct antagonism between biomass for food and energy uses but indirect competition for land, thus, only not competitive for food production should be taken into consideration for energy purposes [[16], [17], [18]]. On this basis, agricultural residues have recently gained attention as bioenergy feedstocks due to various benefits they provide such as thelow-cost of material, the absence of antagonism as a food source and the high annual production [19,20]. Agricultural residues are usually obtained during harvesting of annual crops and include primary residues, such as leaves, stalks, and stover, and secondary residues that remain after processing into a valuable resource [21]. As stated by Avcioğlu et al. [22], agricultural residues are strongly related to crop yield and constitute a certain percentage of the total agicultural crop production. In particular, the utilization of agricultural residues obtained from dedicated annual energy crops is of considerable interest due to fact that these crops positively contribute to rural development, enable crop production diversification and represent an alternative income for farmers [9,23]. Moreover, when grown on marginal lands, these crops are not suited for competition with rotational arable food crops and produce acceptable yields [24].

In Italy, maize (*Zea mays* L.), rapeseed (*Brassica napus* var. *oleifera* L.), soya (*Glycine max* (L.) Merr.) and sunflower (*Helianthus annuus* L.) are the most grown dedicated annual crops for energy purposes and production is mainly located in central-northern regions. In areas of Southern Italy, these species are not widely grown because of a series of agronomic and economic reasons, the cultivation of cereal and legume crops thus prevailing. However, previous studies have demonstrated that most oilseed crops can be successfully grown into cereal-based cropping systems, showing great tolerance to the climate and soil characteristics of these areas, exhibiting reduced needs for agricultural inputs and producing good seed and oil yields [[25], [26], [27]]. In recent years, Ethiopian mustard (*Brassica carinata* A. Braun) and safflower (*Carthamus tinctorius* L.) have assumed increasingly greater importance for energy purposes in southern Italian regions, providing numerous advantages for farmers as confirmed by several studies [[26], [27], [28], [29], [30], [31]]. Ethiopian mustard is a food oilseed crop but its oil, when extremely rich in erucic and linoleic acids, is mainly grown for biodiesel generation [32,33]. In contrast, safflower is largely grown for the high nutritional properties of the oil due to abundant levels of oleic acid [34,35], although some studies have investigated the use of the oil for biodiesel production [36,37]. It is well known that these species, especially Ethiopian mustard, produce a significant amount of agricultural residues per annum; however, there is little or no information concerning their use as a bioenergy source [38,39]. In general, reasons for this apparent disinterest are mainly related to a lower energy potential compared to forestry residues, and to seasonal production, which can lead to uncertain biomass supplies, as stated by Gravalos et al. [40].

Although these relatively minor benefits, farmers in the South Mediterranean who persue circular economy and sustainability goals are interested in the energetic valorisation of agricultural residues mainly for combustion purposes or biogas production as alternative to the method of burying residues. In this context, the estimation of the annual yield of agricultural biomass and the knowledge of the energetic characteristics of the residues are fundamental for on-farm bioenergy production. Finally, considering that the high morphological heterogeneity of agricultural residues can hinder their utilization in thermal systems, transformation into pellet form could enable standardization of the residues and successful use in combustion plants.

This study permits to cover a gap in knowledge regarding the agricultural residues properties as well as energy capacities of Ethiopian mustard and safflower in the Mediterranean region that is needed for possible renewable biomass exploitation. The lack of studies focusing on energetic valorisation of agricultural residues of these species in this region requires, in fact, a deeper investigation in order to determine their energetic potential and give responses to farmers.

The aims of this study were: i) to determine the yield performances of the two species under rainfed conditions; ii) to determine the energetic characteristics of agricultural residues and pellets; iii) to evaluate the energy potential of these residues; iv) to analyse the profitably of Ethiopian mustard and safflower cultivation.

## 2 Materials and methods

### 2.1 Test site

Trials were carried out at the “Calogero Amato Vetrano” Agricultural Technical Institute, located in Sciacca, in the South-West of Sicily (Italy) (37°30′43″N,13°07′32,08″E; 110 m a.s.l.), in two consecutive growing seasons (2017–2018 and 2018–2019). The soil type in the area is sandy clay loam and is classified as Aric. Regosol (USDA classification). The study location has a temperate-warm climate with dry summers and mild winters in accordance with the Köppen–Geiger classification [41]. Taking into consideration the time series 2000–2020, the annual temperature is 18.6 °C while the annual rainfall is 500 mm, on average.

### 2.2 Weather data

A weather station owned by Sicilian Agro-Meteorological Information Service [42] was used to record rainfall and temperatures data. The station was situated 500 m from the Ethiopian mustard and safflower experimental fields and was equipped with various instruments and sensors to measure weather conditions and patterns. In this study, a rain gauge and thermometer were exploited in order to collect rainfall and maximum and minimum temperature data, respectively.

### 2.3 Plant material

Ethiopian mustard seeds were previously provided by the Plant Gene Resources of Canada (PGRC). A total of 20 accessions (CN code) were assessed and more than 50 % of them were Ethiopian in origin (Table 1). Regarding safflower, seeds were instead provided by the Western Regional Plant Introduction Station (WRPIS) of the United States Department of Agriculture (USDA). A total of 19 spiny and non-spiny accessions (PI code) were tested. More than 50 % of the accessions were Chinese in origin (Table 2).

**Table 1:** List of 20 Ethiopian mustard accessions grown during the 2017–2018 and 2018–2019 growing seasons and their area of origin.

**Accession code**	**Name**	**Origin**
CN 101616	PAK 85490	Pakistan
CN 101661	R 3535	Ethiopia
CN 101662	SRS 2202	n.a.^a^
CN 101663	SRS 2203	n.a.
CN 101664	SRS 2204	n.a.
CN 101665	SRS 2205	n.a.
CN 101666	SRS 2206	n.a.
CN 101667	SRS 2207	n.a.
CN 101625	S-23	Ethiopia
CN 101632	S-73	Ethiopia
CN 101633	Awassa population	Ethiopia
CN 101641	PI 194903	n.a.
CN 101683	1977 UC Row 1283	Ethiopia
CN 101684	1977 UC Row 3228	Ethiopia
CN 101685	1977 UC Row 3229	Ethiopia
CN 101686	1977 UC Row 3231	Ethiopia
CN 101687	1977 UC Row 3232	Ethiopia
CN 101688	1977 UC Row 3233	Ethiopia
CN 101689	1977 UC Row 3234	Ethiopia
CN 101698	SRS 3030	n.a.

a not available.

**Table 2:** List of 19 safflower accessions grown during the 2017–2018 and 2018–2019 growing seasons and their area of origin.

**Accession code**	**Name**	**Spiny/spineless**	**Origin**
PI 199952	BJ-763	Spiny	India
PI 251267	BJ-1124	Spineless	Jordan
PI 405961	BJ-2058	Spineless	Iran
PI 537617	1023	Spineless	United States
PI 537625	1031	Spineless	United States
PI 599253	S-317	Spiny	United States
PI 613528	80/635/1S	Spiny	China
PI 638539	Lesaf 487	Spiny	Canada
PI 537110	Quiriego 88	Spiny	Mexico
PI 537619	1025	Spiny	China
PI 653157	Dunhuang Honhua	Spiny	China
PI 653158	Zhangye Youci	Spineless	China
PI 653161	Jimusaer Honhua	Spineless	China
PI 653165	BJ-110	Spiny	China
PI 653184	Bayanzaoer Honhua	Spineless	China
PI 653192	BJ-365	Spiny	China
PI 653193	Bachu Honhua	Spineless	China
PI 653194	Jimusaer Youci	Spineless	China
PI 653219	XJ-072	Spineless	China

### 2.4 Main cultivation practices

A randomized complete block design with three replications [43] was adopted for the tests during the growing seasons. The experimental plot measured 14 m^2^ (5 m × 2.8 m) for Ethiopian mustard plants and 13.5 m^2^ (4.5 m × 3 m) for safflower plants. *Triticum durum* Desf. was the preceding crop. Concerning seedbed preparation, through conventional tillage, soil was ploughed at a depth of 35–40 cm and subsequently harrowed.

With regard to Ethiopian mustard, sowing was planned on November 15, 2017 and on November 19, 2018, adopting a density of 75–80 viable seeds m^−2^ and row spacing of 35 cm. In pre-sowing, 100 kg ha^−1^ of phosphorus (P) fertilizer was applied; 120 kg ha^−1^ of nitrogen (N) fertilizer was used of which 50 kg ha^−1^ during sowing and 70.0 kg ha^−1^ prior to stem elongation. In the case of safflower, the sowing dates were November 13, 2017 and November 16, 2018, respectively. A density of 50 viable seeds m^−2^ was employed and row spacing was 50 cm. In pre-sowing, 80 kg ha^−1^ of P fertiliser was applied; 100.0 kg ha^−1^ of N fertiliser was used of which 50 kg ha^−1^ at sowing time and 50 kg ha^−1^ at the start of stem elongation.

Ethiopian mustard and safflower plants were cultivated under rainfed condition. Dicotyledonous weeds were mechanically managed while graminaceous weeds were controlled applying fluazifop-p-butyl 13.4 % at a rate of 1.0 L ha^−1^. Concerning pest control, dimethoate 98.0 % at a rate of 1.50 L ha^−1^ was applied at the beginning of the flowering stage. At seed ripening, when the seed moisture content was less than 16.0 % (Ethiopian mustard) and 12.0 % (safflower), harvest was carried out. This practice was made by using a combine harvester equipped with a wheat-cutting bar. The harvesting activities occurred between the 3rd 10-day period of June and the 1st 10-day period of July during both growing seasons.

### 2.5 Plant growth analysis and biomass yield measurements

The growth stages of Ethiopian mustard and safflower plants were documented in accordance with Lancashire et al. [44] and Flemmer et al. [45]. The codes of some growth stages were common between the species while other codes were different.

Germination stage (BBCH scale code = 00–09) was measured for both species from sowing of the dry seed to cotyledon emergence. Stem elongation stage (BBCH scale code = 30–39) was detected from the beginning of stem elongation to more visibly extended internodes. Flowering stage (BBCH scale code = 60–69) was determined from the beginning to the end of flowering.

With regards Ethiopian mustard, ripening stage (BBCH scale code = 80–89) was measured until full ripening while senescence stage (BBCH scale code = 97–99) was recorded from the time of the plants becoming dry to the harvested product. In the case of safflower, fruit ripening stage (BBCH scale code = 81–89) was determined when the capitula were ready for harvest while senescence stage (BBCH scale code = 91–97) was detected when most capitula turned yellow.

The amount of growing degree-days (GDDs) accumulated by Ethiopian mustard and safflower plants was used to analyse the growth stages of the two crops. For each growth stage, daily GDDs were calculated with equation (1) provided by McMaster and Wilhelm [46] (1997):

(1) $$GDD=\frac{\left(T_{\max}+T_{\min}\right)}{2}-T_{base}$$

where: T_max_ and T_min_ are daily maximum and minimum air temperatures and T_base_ is the base temperature. Values of 4 and 5 °C were used as the base temperature for the Ethiopian mustard and safflower plants [47], respectively.

At harvesting stage, crop residue and seed yields were detected on a harvest area of 7 m^2^ for both crops. Plant height, number of branches and 1000-seed weight (TSW) were measured on a sample of 10 plants per plot. With regards to Ethiopian mustard, number of siliquae per plant, siliqua length and number of seeds per siliqua were also recorded whilst, in the case of safflower, number of capitula per plant was determined.

Samples of each vegetable fraction were dried at 60 °C in an oven until constant weight to estimate the dry biomass of the aboveground plant parts.

### 2.6 Energetic characteristics of agricultural residues and pellet

Agricultural residues of Ethiopian mustard and safflower accessions consisted of stover that was collected from the experimental fields and subsequently stored. Energetic characterisation of the raw material was carried out by evaluating moisture and ash content, higher heating value (HHV), following the Italian and international regulations.

Moisture content was measured in line with UNI EN 14774–2:2009 regulation [48], adopting a forced ventilation oven. Ash content was obtained following the UNI EN 14775:2010 regulation [49]. Dry samples were placed in a muffle furnace at 500 °C for approx. 2 h with a temperature gradient of 4 °C min^−1^. HHV for the ash-free dry matter, however, was calculated in accordance with UNI EN 14918:2010 regulation [50], exploiting a Berthelot-Mahler bomb calorimeter. Lower heating value (LHV) of agricultural residues was determined from the HHV by subtracting the heat of water vaporization in the product, as equation (2) shows [51]:

(2) $$LHV=HHV-2.441\times \left(MC+9\times H_{cont}\right)$$

where MC is the wet basis moisture content, the constant 2.441 is the latent heat of water vaporization in MJ kg^−1^ at 25 °C and H_cont_ is the hydrogen content of agricultural residues.

Agricultural residues were subsequently proved in pellet-making and the end product was evaluated both in energetic and physical terms by calculating the ash content, HHV and mechanical durability (MD). The energetic characterisation of the pellet was carried out following regulation UNI EN-14961–2:2011 [52]. Pellets were obtained using shredded residues which were fed into a pellet machine. MD, whose definition has been clearly reported by Gil et al. [53], was measured using the Lignotester New Holmen Tester TekPro and calculated with equation (3) provided by UNI EN-15210–1:2009 [54]:

(3) $$MD=\frac{\text{MA}}{ME}\times 100$$

where MA is the mass of the pellet before mechanical shaking and ME the is mass of pellet after mechanical treatment. Ash content and HHV values of the pellet were obtained exploiting the same methodological procedures as for residues.

### 2.7 Theoretical biomass potential

Theoretical biomass potential (TBP) can be defined as the total annual production of agricultural, forestry and other residues in a specific region [55]. In this study, it was determined with reference only to total annual production of biomass provided by agricultural residues of the Ethiopian mustard and safflower accessions. It was exploited to calculate the total quantity of agriculture residues for Ethiopian mustard and safflower accessions grown in the study area. The losses of biomass from both storage and transport sites were not included in the analysis. TBP was calculated using equation (4) provided by Avcioğlu et al. [22]:

(4) $$TBP=CP\times RPR\times \left[\frac{100-M}{100}\right]$$

where CP is the amount of seed obtained per year as tons per hectare; RPR is the ratio between agricultural residue and seed yield (SY) produced per year, and the result is dimensionless; M is the relative moisture content expressed in percentage terms. For each crop, the value of TPB was expressed as average of all accessions.

### 2.8 Theoretical biomass energy potential

Theoretical energy potential (TEP) represents the total annual production of energy produced by dry residues. In this study, it was adopted to determine the energy potential of agricultural residues obtained by Ethiopian mustard and safflower accessions grown in the study area. TEP was estimated using equation (5) provided by Avcioğlu et al. [22]:

(5) TEP=TBP×LHV

For each crop, the value of TEP was expressed as average of all accessions.

### 2.9 Available biomass energy potential

Available energy potential (AEP) is defined as the energy content of biomass that can be economically and technically harvested [50]. The AEP of agricultural residues for each crop was calculated by using equation (6) in accordance with Avcioğlu et al. [22]:

(6) AEP=TEP×A

where A is the available residue ratio as a percentage. It was calculated as ratio between agriculture residue and total biomass yields (TBYs). For each crop, the value of AEP was expressed as average of all accessions.

### 2.10 Profit analysis of ethiopian mustard and safflower cultivation

In Italy, the production of biomass for energy purposes primarily originates within farms which are specialized in monocultures and/or those whose production is diversified into a variety of goods and services. These agro-industrial producers can achieve real economies of scale or scope, as affirmed by Sgroi [56]. Consequently, profit analysis of the biomass production should be based on the application of concepts and criteria of microeconomics [57,58]. To determine the economic feasibility, we hypothesized to consider a farm that produced two main goods for energy production: Ethiopian mustard (Q1) and safflower (Q2). It was assumed that the production of Q1 and Q2 did not influence the market prices (P1 and P2) of the two crops. The gross income of their cultivation was calculated by subtracting the variable costs from the production value. Each variable cost item was given by the sum of the costs of materials originating outside the farm and the hours of work (man and machine) used to carry out the various cultivation practices (sowing, tillage, fertilisation, weeds and pests control, seed harvesting, and crop residue harvesting). The production value was given by the sum of the revenues obtained from the sale of seeds and agricultural residues; for each crop, the market price of seed and agricultural residue was multiplied for the amount produced per hectare. The gross income represents the margin contribution of the crop considered to the management of the company. In the economic literature, gross income can be useful for comparing two crops that have the same fixed resource needs. Concerning the costs, they were estimated in accordance with current and local market prices. In the case of production value of the two crops, the values provided by the Venetian Payment Agency [59] were taken into consideration.

### 2.11 Statistical analyss

The statistical analysis was carried out with the use of the MINITAB software for Windows (version 19.0, USA). The analysis of variance (ANOVA) was adopted to test the effects of genotype and year on the morphological and productive traits of Ethiopian mustard and safflower plants and energetic parameters of agricultural residues and pellet. When ANOVA showed statistically different means, the Tukey test was used to separate means (P ≤ 0.01). All the representative values concerning various energetic parameters, theoretical biomass potential, theoretical energy potential and available energy biomass of the two crops were presented using mean ± standard error calculations.

## 3 Results

### 3.1 Analysis of rainfall and temperature in the study area

**Fig. 1:**
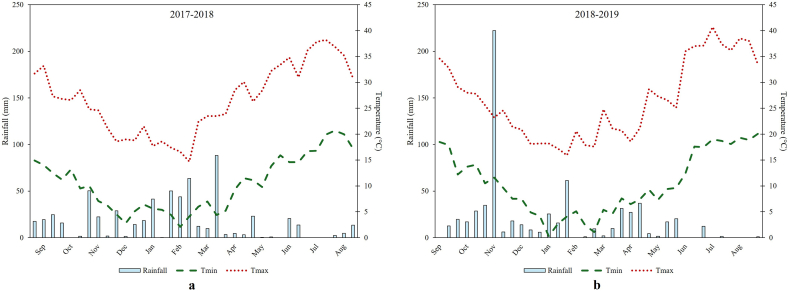
Rainfall and temperature trends during the test period.

In the two growing seasons, average minima and maxima temperatures were similar and consistent with ten-year average temperatures (Fig. 1a and b). Temperatures decreased from November to February and increased up to June, when ripening occurred for both species. With reference to the plant growth cycle of both species, highest maximum temperatures were observed in the first 10-day period of July 2018 (36.2 °C) and 2019 (40.7 °C), when harvest occurred. Lowest minimum temperatures were determined in the second 10-day period of February 2018 (4 °C) and 2019 (2.4 °C), when leaf development began. The two crops did not suffer frost damage during winter, although minima temperatures falling below 4 °C in each growing season (Fig. 1). Ethiopian mustard and safflower plants showed high drought tolerance and did not suffer heat damage when temperatures rose above 30 °C during summer.

Total annual rainfall levels were different in the two years. In particular, the 2017–2018 growing season was the rainiest (466.2 mm). Rainfall pattern was irregular during the seasons and this affected the water needs of the crops. Rainy days were, in fact, more distributed in winter (80 %) than in spring (Fig. 1). However, the more uniform distribution of rainfall during the first growing season contributed to increase the soil water availability over a longer period in comparison with the second growing season. This strongly influenced the duration of the vegetative and reproductive phases of the two crops. Between May and July, when ripening and senescence stages occurred, rainfall levels were higher than 50 mm in both growing seasons.

### 3.2 Ethiopian mustard

#### 3.2.1 Plant growth stages

In the first growing season, the growth cycle was slightly shorter by two days compared to the second season due to differing air temperatures during germination and ripening stages (Table 3). In 2017–2018, lower minima temperatures at germination stage slowed down seedling emergence while higher minimum temperatures at ripening stage determined faster silique maturation starting from May. The contrary occurred, however, in terms of minimum temperatures in 2018–2019. The effect of rainfall on the duration of the ripening stage was the same in both years. From May to June, rainfall ranged from 58.1 mm (2017–2018) to 51.2 mm (2018–2019) and did not determine evident stress conditions in the plants.

**Table 3:** Duration and cumulative growing degree days required from various growth stages of Ethiopian mustard during two consecutive growing seasons. For each growth stage, average value of the 20 accessions is shown.

**Species**	**Growing season**	**Duration (days)**				
		Germination	Stem elongation	Flowering	Ripening	Senescence
*Brassica carinata*	2017–2018	30	143	171	234	246
	2018–2019	25	140	178	236	248
		**GDD (°C day)**				
		Germination	Stem elongation	Flowering	Ripening	Senescence
*Brassica carinata*	2017–2018	265	1179	1577	2735	3014
	2018–2019	261	1133	1581	2747	3015

When comparing the two years, germination stage was observed within 30 days in the 2017–2018 growing season, and within 25 days in the 2018–2019 growing season, on average. Stem elongation stage lasted 141 days from the sowing date. Flowering stage was recorded earlier in the first growing season. Accessions CN 101661 and CN 101687 achieved 50 % open-flower phase earlier than the others in the two years (data not shown). Fruit ripening stage was detected within 234 days in the 2017–2018 growing season, and 236 days in the 2018–2019 growing season, on average. In some accessions, it occurred when flowering stage had not yet reached 50 %. Senescence stage occurred when maxima air temperatures were receorded upper 0° than 3C and rainfall was lacking. Plants were dry and siliques became brown. Ethiopian mustard accessions ended their growth cycle before the third 10-day period of July in the first season and within the third 10-day period of July in the second season.

With regard to GDDs, accessions accumulated 3014 GDDs in the 2017–2018 growing season, and 3015 in the 2018–2019 growing seasons to complete their growth cycle. No great differences were found among the accessions regarding GDDs over the two years.

#### 3.2.2 Morphological and yield component performance

Genotype and year determined significant differences in all morphological and productive traits in the study (Table 4). Results of ANOVA also showed that the interaction between the main factors was significant for all traits. All the *p*-values were found highly significant which indicated low coefficient of variation values (Table S1).

Plant height ranged from 160.80 cm (1st-growing season) to 158.08 cm (2nd-growing season). Ethiopian mustard accessions revealed high variability and obtained an average value of 159.42 cm. In the two growing seasons, CN 101698 (190.10 cm) produced the highest performance in plant height while CN 101663 (134.83 cm) had the lowest average values. Concerning the number of branches per plant, the highest average value was recorded in the first year (12.47). CN 101664 (11.74) had the highest value whilst CN 101641 (7.27) obtained the lowest value for this morphological trait, on average.

The number of siliques per plant showed average values of 430.51 (1st growing season) and 421.06 (2nd growing season). This trait proved significantly affected by accessions. CN 101698 and CN 101661 obtained the highest (647.70) and lowest (286.73) average values. Silique length and number of seeds per silique recorded the highest average values in the first growing season. Regarding silique length, the accessions did not show high variability and this trait ranged from 4.66 cm (CN 101661) to 3.85 cm (CN 101665).

TSW showed significant differences in the study period, recording the highest average value (3.71 g) in the second growing season. Across accessions, TSW ranged between 3.82 g (CN 101662) and 2.86 g (CN 101666, CN 101689), with an average value of 3.34 g.

Concerning the yield parameters, the best performances for SY (2.99 t ha^−1^), ARY (7.93 t ha^−1^) and TBY (10.92 t ha^−1^) were produced, on average, during the first growing season. Only 7 accessions obtained an average SY which was higher than 3.00 t ha^−1^, whilst average values for 3 accessions were lower than 2.50 t ha^−1^. CN 101698 produced the highest ARY (10.66 t ha^−1^) while, on average, CN 101625 provided the lowest values (6.06 t ha^−1^). Average TBYs for the Ethiopian mustard accessions was 10.71 t ha^−1^; however, high variability was observed for this yield trait.

The year-by-genotype interaction relevantly affected all yield components and showed similar values for the two growing seasons. TSW, particularly, was found on average to be highest in the second growing season. The highest SY were found in the first growing season. Regarding agricultural residues, the best performance was obtained during the second growing season.

**Table 4:** Morphological and yield parameters of the 20 Ethiopian mustard accessions during two consecutive growing seasons.

**Main variables**	**Plant height (cm)**	**Number of branches (n)**	**Number of siliquae (n)**	**Siliqua lenght (cm)**	**TSW (g)**	**Seed yield (t ha^-1^)**	**Agricultural residue yield (t ha^-1^)**	**Total biomass yield (t ha^-1^)**
Year (Y)								
2017–2018	160.80 a	12.47 a	430.51 a	4.57 a	2.90 b	2.99 a	7.93 a	10.92 a
2018–2019	158.08 b	6.08 b	421.06 b	3.94 b	3.71 a	2.81 b	7.62 b	10.44 b
Genotype (G)								
CN 101616	165.00 e	10.30 abc	432.70 g	4.56 ab	3.82 a	2.27 k	6.75 k	9.02 jk
CN 101661	163.80 ef	9.20 abc	286.73 n	4.66 a	3.72 ab	2.89 ef	8.65 d	11.54 cd
CN 101662	164.70 e	8.56 abc	368.93 j	4.50 abc	3.95 a	2.50 ij	7.29 ij	9.79 i
CN 101663	134.83 k	9.94 abc	429.13 g	4.22 abc	3.26 cd	2.58 hi	7.08 j	9.67 i
CN 101664	170.56 c	11.74 a	584.36 c	4.01 abc	3.74 ab	3.42 bc	7.86 gh	11.29 de
CN 101665	151.20 h	9.64 abc	302.60 l	3.85 c	3.27 cd	3.09 d	8.27 ef	11.35 de
CN 101666	141.20 j	7.66 abc	396.73 i	3.96 bc	2.85 e	2.87 ef	6.27 l	9.13 j
CN 101667	167.46 d	8.60 abc	432.01 g	3.91 bc	3.66 ab	3.55 ab	10.08 b	13.64 a
CN 101625	147.40 i	10.56 abc	292.43 m	4.46 abc	2.98 de	2.97 de	6.06 l	9.04 jk
CN 101632	150.83 h	7.50 bc	358.27 k	4.58 ab	3.16 cde	2.35 k	6.73 k	9.08 j
CN 101633	173.40 b	9.77 abc	431.96 g	4.33 abc	3.08 de	2.39 jk	7.45 i	9.84 hi
CN 101641	163.90 ef	7.27 c	241.56 o	4.53 ab	3.31 cd	2.64 gh	7.59 hi	10.20 gh
CN 101683	161.90 fg	8.20 abc	576.43 d	4.04 abc	3.42 bc	3.07 d	8.04 fg	11.11 e
CN 101684	149.40 hi	9.46 abc	549.47 e	4.15 abc	3.08 de	3.39 c	9.01 c	12.40 b
CN 101685	149.13 hi	7.77 abc	393.80 i	4.04 abc	3.25 cd	3.36 c	7.08 j	10.45 fg
CN 101686	160.70 g	9.33 abc	403.10 h	4.39 abc	3.03 de	2.87 ef	8.53 de	11.39 de
CN 101687	164.10 ef	9.23 abc	491.87 f	4.39 abc	3.16 cde	2.56 hi	6.11 l	8.67 k
CN 101688	149.30 hi	11.47 ab	301.40 l	4.34 abc	3.43 bc	2.77 fg	7.83 gh	10.60 f
CN 101689	169.67 cd	10.63 abc	594.50 b	4.12 abc	2.87 e	3.64 a	8.20 f	11.84 c
CN 101698	190.10 a	8.70 abc	647.70 a	4.13 abc	3.07 de	2.91 ef	10.66 a	13.57 a
Source of variation (p-value)								
Y	0.00 ∗∗	0.00 ∗∗	0.00 ∗∗	0.00 ∗∗	0.00 ∗∗	0.00 ∗∗	0.00 ∗∗	0.00 ∗∗
G	0.00 ∗∗	0.00 ∗∗	0.00 ∗∗	0.00 ∗∗	0.00 ∗∗	0.00 ∗∗	0.00 ∗∗	0.00 ∗∗
Y × G	0.00 ∗∗	0.00 ∗∗	0.00 ∗∗	0.00 ∗∗	0.00 ∗∗	0.00 ∗∗	0.00 ∗∗	0.00 ∗∗

Means followed by the same letter are not significantly different for p ≤ 0.01 according to Tukey's test. ∗∗ significant at the 0.01 probability level.

#### 3.2.3 Energetic characteristics of agricultural residues and pellet

The main factors produced significant differences for all traits in the study (Table 5). Results of ANOVA revealed that all energetic characteristics of agricultural residues were not significantly influenced by the year-by-genotype interaction. On the contrary, this interaction determined significant variations for all energetic parameters regarding pellets.

**Table 5:** Main energetic parameters of agricultural residue and pellet of the 20 Ethiopian mustard accessions during two consecutive growing seasons.

**Main variables**	**Moisture content (%)**	**Ash content (%)**	**HHV (MJ kg^-1^)**	**LHV (MJ kg^-1^)**	**Ash content (%)**	**HHV (MJ kg^-1^)**	**Mechanical durability (%)**
	**Agricultural residue**				**Pellet**		
Year (Y)							
2017–2018	15.07 ab	6.67 b	13.65 a	11.83 a	6.82 a	16.64 a	73.68 a
2018–2019	15.22 a	6.74 a	13.52 b	11.69 b	6.70 b	16.25 b	71.61 b
Genotype (G)							
CN 101616	16.23 b	7.27 c	13.99 a	12.15 ab	6.41 i	13.92 j	72.45 e
CN 101661	15.06 j	6.88 e	13.35 gh	11.51 kl	5.30 m	16.31 i	74.63 ab
CN 101662	15.21 i	5.69 k	13.44 efg	11.62 hij	7.05 e	16.48 h	74.59 abc
CN 101663	15.34 h	6.39 hi	13.52 de	11.68 ghi	7.39 d	16.55 efgh	72.16 e
CN 101664	15.51 g	7.15 d	13.60 d	11.77 fg	6.43 i	16.59 def	72.30 e
CN 101665	15.67 ef	7.10 d	14.07 a	12.23 a	6.96 f	17.13 a	74.24 bc
CN 101666	15.81 d	6.61 f	13.79 bc	11.95 cd	7.46 d	16.76 c	74.83 a
CN 101667	15.97 c	7.84 a	13.87 b	12.01 c	6.18 k	16.83 bc	75.12 a
CN 101625	15.73 def	5.53 l	13.73 c	11.89 de	6.25 j	16.58 defg	74.00 c
CN 101632	11.71 n	7.34 c	13.57 d	11.82 ef	7.90 a	16.49 gh	72.05 ef
CN 101633	16.55 a	7.64 b	13.98 a	12.12 b	5.34 m	16.89 b	75.12 a
CN 101641	15.74 de	6.46 gh	13.46 ef	11.62 hij	7.68 c	16.59 def	70.36 i
CN 101683	13.01 l	6.17 j	13.29 h	11.52 jkl	6.56 h	16.28 i	73.35 d
CN 101684	14.62 k	6.29 i	13.37 fgh	11.54 jkl	6.90 f	16.27 i	70.28 i
CN 101685	15.63 f	7.31 c	13.45 efg	11.62 hij	7.79 b	16.51 fgh	71.25 gh
CN 101686	16.63 a	5.02 m	13.53 de	11.66 ghi	6.48 i	16.61 de	70.68 hi
CN 101687	14.62 k	6.63 f	13.52 de	11.71 gh	6.70 g	16.65 d	71.94 ef
CN 101688	15.67 ef	7.13 d	13.43 efg	11.59 ijk	7.93 a	16.28 i	71.55 fg
CN 101689	16.19 b	6.98 e	13.29 h	11.44 l	6.66 g	16.60 def	70.63 i
CN 101698	11.96 m	6.55 fg	13.46 ef	11.70 gh	5.85 l	16.55 defgh	71.32 g
Source of variation (p-value)							
Y	0.00 ∗∗	0.00 ∗∗	0.00 ∗∗	0.00 ∗∗	0.00 ∗∗	0.00 ∗∗	0.00 ∗∗
G	0.00 ∗∗	0.00 ∗∗	0.00 ∗∗	0.00 ∗∗	0.00 ∗∗	0.00 ∗∗	0.00 ∗∗
Y × G	1.00 n.s.	1.00 n.s.	1.00 n.s.	1.00 n.s.	0.00 ∗∗	0.00 ∗∗	0.00 ∗∗

Means followed by the same letter are not significantly different for p ≤ 0.01 according to Tukey's test. ∗∗ significant at the 0.01 probability level; n.s. not significant.

In the case of agricultural residues, moisture content ranged from 16.63 % (CN 101686) to 11.71 % (CN 101632) with an average of 15.11 %. The highest average ash content (7.84 %) was observed in CN 101667 whilst the lowest (5.02 %) in CN 101686. The highest performance of HHV and LHV were found in the second growing season. Most accessions had similar average HHV and LHV values. CN 101665 and CN 101632 obtained the highest average HHV (14.07 MJ kg^−1^) and LHV (11.70 MJ kg^−1^) values. In the case of C-H-N fractions, the residues were found to have the highest C content and the lowest N content as a percentage of dry matter (data not shown).

Regarding the pellets, the ash content ranged from 6.82 % (1st growing season) to 6.70 % (2nd growing season). Accessions had high variability for this parameter and obtained an average value of 11.28 %. The highest performance in HHV was produced in the first year (16.64 MJ kg^−1^). Pellets obtained from agricultural residues CN 101665 (17.13 MJ kg^−1^) and CN 101633 (16.89 MJ kg^−1^), on average, produced the highest HHV values. Mechanical durability of the pellets ranged from 75.83 % (CN 101666) to 70.36 % (CN 101641). All accessions showed average mechanical durability values above 70.00 %. In general, only 2 accessions produced the highest average HHV and mechanical durability values, and the lowest average ash content value.

Focusing on year-by-genotype interactions, average HHV and mechanical durability for pellets were highest in the first growing season.

### 3.3 Safflower

#### 3.3.1 Plant growth stages

In the two growing seasons, safflower accessions had a similar growth cycle length, with an average duration of 220 days in the 2017–2018 season and 222 days in the 2018–2019 season (Table 6). Air temperature and rainfall did not produce evident changes in the duration of the main growth stages, similar to Ethiopian mustard. Germination stage occurred within 18 days in the 2017–2018 growing season and within 16 days in that of 2018–2019, based on climate conditions. Stem elongation stage lasted an average of 79 days from the sowing date. Flowering stage was recorded earlier in the second growing season (158 days). When comparing the accessions, 4 genotypes reached fruit ripening stage earlier than others in the two years (data not shown). Flowering stage occurred within 203 days in the 2017–2018 growing season and 207 days in the 2018–2019 season, on average. Senescence stage was observed when maximum air temperature increased to 30 °C and rainfall was not detected. Plants were dry and the capitula turned brown. The accessions ended their cycle within the third 10-day period of June in both growing seasons.

**Table 6:** Duration and cumulative growing degree days required from various growth stages of safflower during two consecutive growing seasons. For each growth stage, average value of the 19 accessions is shown.

**Species**	**Growing season**	**Duration (days)**				
		Germination	Stem elongation	Flowering	Ripening	Senescence
*Carthamus tinctorius*	2017–2018	18	82	160	203	220
	2018–2019	16	77	158	207	222
		**GDD (°C day)**				
		Germination	Stem elongation	Flowering	Ripening	Senescence
*Carthamus tinctorius*	2017–2018	159	601	1235	1891	2205
	2018–2019	166	537	1162	1806	2142

The accessions accumulated on average 2205 GDDs and 2142 GDDs in the first and second growing seasons, respectively, to complete their growth cycle.

#### 3.3.2 Morphological and yield component performance

The main fixed factors produced significant differences for all morphological and productive traits of safflower. Furthermore, the year-by-genotype interaction determined significant variations for all traits in the study (Table 7). All the *p*-values were found highly significant which indicated low coefficient of variation values (Table S2).

**Table 7:** Morphological and yield parameters of the 19 safflower accessions during two consecutive growing seasons.

**Main variables**	**Moisture content (%)**	**Ash content (%)**	**HHV (MJ kg^-1^)**	**LHV (MJ kg^-1^)**	**Ash content (%)**	**HHV (MJ kg^-1^)**	**Mechanical durability (%)**
	**Agricultural residue**				**Pellet**		
Year (Y)							
2017–2018	11.35 a	4.57 a	14.27 a	12.55 a	5.16 a	17.33 a	75.50 a
2018–2019	11.18 a	4.49 b	14.23 a	12.51 a	4.93 b	17.19 b	75.18 b
Genotype (G)							
PI 199952	11.87 a	4.66 d	14.44 cdef	12.68 cdef	4.31 l	17.33 gh	75.42 e
PI 251267	10.93 bcd	5.03 a	14.21 defg	12.47 defg	4.35 l	17.25 i	76.83 a
PI 405961	11.08 abcd	3.26 k	14.83 bc	13.14 bc	5.04 f	17.82 d	76.24 c
PI 537617	11.82 ab	4.19 i	14.06 efgh	12.35 efgh	5.59 b	16.97 l	76.52 b
PI 537625	11.25 abcd	5.04 a	14.47 cde	12.75 cde	5.54 b	17.33 gh	75.27 f
PI 599253	11.47 abcd	4.50 f	15.33 ab	13.60 ab	4.96 d	18.19 c	75.27 f
PI 613528	11.32 abcd	4.31 h	13.58 h	11.86 h	4.78 ij	16.58 n	75.10 g
PI 638539	11.47 abcd	4.82 b	14.39 cdef	12.66 cdef	4.58 k	17.36 g	75.93 d
PI 537110	11.24 abcd	4.85 b	14.68 cd	12.99 cd	5.47 c	17.07 k	74.23 ij
PI 537619	10.92 bcd	4.74 c	14.21 defg	12.49 defg	4.61 k	17.63 f	74.67 h
PI 653157	10.83 d	5.51 a	15.67 a	13.99 a	4.77 j	17.15 j	75.83 d
PI 653158	11.47 abcd	4.75 c	12.86 i	11.16 i	5.58 b	18.72 b	75.11 g
PI 653161	11.03 abcd	4.81 b	14.36 cdef	12.67 cdef	5.29 d	15.83 o	75.14 fg
PI 653165	11.14 abcd	3.60 j	14.80 bcd	13.10 bc	4.86 h	17.30 hi	74.35 i
PI 653184	11.27 abcd	4.19 i	13.67 gh	11.97 gh	4.82 i	17.72 e	74.10 j
PI 653192	11.62 abcd	4.60 e	12.85 i	11.13 i	5.07 ef	16.53 n	75.86 d
PI 653193	11.74 abc	4.67 d	13.85 fgh	12.12 fgh	5.09 e	15.63 p	74.65 h
PI 653194	10.85 cd	4.61 e	15.67 a	13.94 a	5.50 c	16.77 m	75.93 d
PI 653219	10.79 d	4.44 g	12.81 i	11.09 i	5.72 a	18.81 a	75.07 g
Source of variation (p-value)							
Y	0.030 n.s.	0.00 ∗∗	0.396 n.s.	0.436 n.s.	0.00 ∗∗	0.00 ∗∗	0.00 ∗∗
G	0.00 ∗∗	0.00 ∗∗	0.00 ∗∗	0.00 ∗∗	0.00 ∗∗	0.00 ∗∗	0.00 ∗∗
Y × G	0.00 ∗∗	0.00 ∗∗	0.00 ∗∗	0.00 ∗∗	0.00 ∗∗	0.00 ∗∗	0.00 ∗∗

Means followed by the same letter are not significantly different for p ≤ 0.01 according to Tukey's test. ∗∗ significant at the 0.01 probability level; n.s. not significant.

Plant height produced average values of 149.64 cm (1st growing season) and 148.41 cm (2nd growing season). Across the accessions, this trait ranged between 174.60 cm (PI 199952) and 119.36 cm (PI 653192), with an average value of 149.02 cm. The accessions showed an appreciable variability in number of branches per plant. On average, PI 599253 obtained the highest value (7.39) whilst PI 251267 (4.69) recorded the lowest value.

The number of capitula was higher in the first growing season (11.05) compared to the second one (10.35). PI 599253 (15.19) and PI 199952 (14.63) obtained the highest performance with respect to an average value of 10.74 for all accessions. The lowest number of capitula (6.68) was found in PI 537110. Regarding TSW, the highest average value (40.60 g) was found in the first growing season. This trait ranged between 57.48 g (PI 251267) and 30.18 g (PI 638539) with an average value of 40.02 g.

The best performance of average SY (1.56 t ha^−1^), ARY (1.18 t ha^−1^) and TBY (2.76 t ha^−1^) were recorded in the first year. Regarding SY, PI 653219 obtained the highest value (2.17 t ha^−1^) whilst PI 65315 recorded the lowest value (0.74 t ha^−1^). PI 251267, on average, produced the highest quantity (1.83 t ha^−1^) of agricultural residues. Only 5 safflower accessions showed ARYs below 1.00 t ha^−1^. The average TBY for the safflower accessions was 2.72 t ha^−1^; however, high variability was observed for this yield trait among the accessions and over the two growing seasons.

The year-by-genotype interaction relevantly influenced all yield components. When considering the two growing seasons, yield values were found to be very similar. The highest average values of SY, ARY and TBY were recorded in the first growing season.

#### 3.3.3 Energetic characteristics of agricultural residues and pellet

Table 8 shows the results of the energetic characterisation of agricultural residues and pellets for safflower accessions.

**Table 8:** Main energetic parameters of agricultural residue and pellet of the 19 safflower accessions during two consecutive growing seasons.

**Main variables**	**Plant height (cm)**	**Number of branches (n)**	**Number of capitula (n)**	**TSW (g)**	**Seed yield (t ha^-1^)**	**Agricultural residue yield (t ha^-1^)**	**Total biomass yield (t ha^-1^)**
Year (Y)							
2017–2018	149.64 a	6.17 a	11.05 a	40.60 a	1.56 a	1.18 a	2.76 a
2018–2019	148.41 b	5.29 b	10.35 b	39.47 b	1.48 b	1.12 b	2.61 b
Genotype (G)							
PI 199952	174.60 a	6.97 b	14.63 a	37.49 i	1.76 f	1.57 d	3.34 d
PI 251267	174.18 a	4.69 m	9.69 def	57.48 a	1.88 d	1.83 a	3.72 b
PI 405961	160.75 bc	6.26 d	12.13 bc	33.96 j	1.69 g	1.27 f	2.97 f
PI 537617	122.95 hi	5.87 fg	10.49 cdef	37.43 i	1.45 i	1.00 i	2.45 i
PI 537625	170.32 a	5.62 ghi	11.24 bcd	32.55 jk	1.77 f	1.44 e	3.20 e
PI 599253	161.15 bc	7.39 a	15.19 a	32.05 jk	1.80 e	1.65 c	3.46 c
PI 613528	144.63 ef	4.06 n	9.86 def	43.02 bcde	1.41 j	1.02 i	2.44 i
PI 638539	134.61 g	6.34 cd	12.53 b	30.18 k	1.37 k	1.05 h	2.42 i
PI 537110	148.18 de	4.94 lm	6.68 g	42.43 bcdef	1.70 g	1.08 h	2.78 h
PI 537619	164.49 b	5.40 ijk	11.29 bcd	42.56 bcdef	2.01 b	1.41 e	3.43 c
PI 653157	170.11 a	5.57 hij	8.89 f	39.26 ghi	1.96 c	1.80 b	3.76 ab
PI 653158	124.41 h	5.19 kl	9.34 ef	41.42 cdefg	0.93 n	0.44 l	1.37 l
PI 653161	122.80 hi	5.90 ef	9.65 def	40.70 defgh	0.74 p	0.57 k	1.30 m
PI 653165	126.18 h	5.82 fgh	10.46 cdef	39.88 fghi	1.03 m	0.55 k	1.59 k
PI 653184	162.58 bc	6.14 de	8.86 f	38.11 hi	1.56 h	1.44 e	3.00 f
PI 653192	119.36 i	5.34 jk	12.61 b	44.68 b	0.80 o	0.42 l	1.23 n
PI 653193	159.54 c	5.37 ijk	10.95 bcde	43.43 bcd	1.79 ef	1.11 g	2.91 g
PI 653194	141.39 f	5.40 ijk	9.42 ef	40.18 efghi	1.14 l	0.67 j	1.82 j
PI 653219	149.35 d	6.60 c	9.33 ef	44.01 bc	2.17 a	1.64 c	3.82 a
Source of variation (p-value)							
Y	0.00 ∗∗	0.00 ∗∗	0.00 ∗∗	0.00 ∗∗	0.00 ∗∗	0.00 ∗∗	0.00 ∗∗
G	0.00 ∗∗	0.00 ∗∗	0.00 ∗∗	0.00 ∗∗	0.00 ∗∗	0.00 ∗∗	0.00 ∗∗
Y × G	0.00 ∗∗	0.00 ∗∗	0.00 ∗∗	0.00 ∗∗	0.00 ∗∗	0.00 ∗∗	0.00 ∗∗

Means followed by the same letter are not significantly different for p ≤ 0.01 according to Tukey's test. ∗∗ significant at the 0.01 probability level.

In the case of agricultural residues, the year factor did not produce significant variations in parameters, except for ash content. In contrast, the genotype factor significantly influenced all the energetic characteristics of residues. ANOVA revealed that the year-by-genotype interaction significantly affected all parameters in the study.

Across the accessions, moisture content ranged from 11.87 % (PI 199952) to 10.79 % (PI 653219), with an average of 11.30 %. Moisture content was lower than 11.00 % in only 5 accessions. Ash content value was higher (4.57 %) in the first growing season compared to the second one (4.49 %). The highest average value of ash content (5.51 %) was found in PI 653157 whilst the lowest (3.36 %) in PI 405961. A small number of safflower accessions were of interest concerning HHV and LHV. In general, average HHV and LHV were 14.30 and 12.35 MJ kg^−1^, respectively. In the case of C-H-N fractions, they had the highest C content and the lowest N content as a percentage of dry matter (data not shown).

Regarding pellets, the genotype and year produced relevant variations for all traits. The year-by-genotype interactions significantly affected all pellet energetic characteristics.

Comparing the two years, the highest average ash content (5.16 %) was recorded in the first growing season. Across the accessions, pellet ash content ranged between 5.72 % (PI 653219) and 4.31 % (PI 199952). Significant variability was observed among the accessions concerning HHV. The best HHV results were observed in pellets obtained from residues PI 653219 (18.81 MJ kg^−1^), PI 653158 (18.72 MJ kg^−1^) and PI 599253 (18.19 MJ kg^−1^). Pellet mechanical durability was very similar in the two years and had an average value of 75.34 % across the accessions. All safflower accessions showed an average value higher than 70.00 %. Observing all accessions, only 1 accession was found to have appreciable HHV and mechanical durability together with low ash content.

Year-by-genotype interaction produced highest average HHV and mechanical durability during the first growing season, in particular.

### 3.4 Biomass energy potential of the two oilseed crops

The agricultural biomass residue properties of Ethiopian mustard and safflower accessions (as average crop perfomance) are reported in Table 9.

**Table 9:** Annually amount of crop production, moisture content, ratio of product residue and lower heating value regarding Ethiopian mustard and safflower. Average ± standard error values are shown.

**Oilseed crops**	**Growing season**	**Residue type**	**Amount of crops product (t ha-1)**^a^	**Moisture content (%)**		**Ratio of product residue**		**Lower Heating Value (MJ kg-1)**	
				Range	Avg.	Range	Avg.	Range	Avg.
*Brassica carinata*	2017–2018	Straw	10.93 ± 0.19	11.60–16.60	15.07 ± 0.17	2.04–3.75	2.65 ± 0.05	11.46–12.36	11.83 ± 0.03
	2018–2019	Straw	10.44 ± 0.19	11.72–16.77	15.22 ± 0.18	2.03–3.82	2.71 ± 0.05	11.33–12.20	11.69 ± 0.03
*Carthamus tinctorius*	2017–2018	Straw	2.76 ± 0.12	10.12–12.79	11.36 ± 0.08	0.47–0.97	0.76 ± 0.02	10.71–15.74	12.55 ± 0.17
	2018–2019	Straw	2.61 ± 0.11	10.03–12.31	11.18 ± 0.08	0.47–0.97	0.76 ± 0.02	10.75–15.74	12.51 ± 0.17

a Oilseed crops production quantities produced annually.

Considering each oilseed crop, crop product amounts were similar over the two growing seasons. Ethiopian mustard produced higher product yields per year (10.68 t ha^−1^) with respect to safflower (2.69 t ha^−1^). Moisture content, ratio of product residue, and LHV varied within a limited range in both crops, highlighting little differences during the study period. The highest ratio of product residue (2.68) was found in Ethiopian mustard.

Theoretical biomass potential, theoretical biomass energy potential and available biomass energy potential were calculated based on stover production estimations for the Ethiopian mustard and safflower experimental fields (Table 10).

**Table 10:** Energy potential of the agricultural biomass residues produced in the Ethiopian mustard and safflower experimental fields during two consecutive growing seasons. Average ± standard error values are shown.

**Oilseed crops**	**Growing season**	**Residue type**	**Amount of crop product (t ha^-1^)**^a^	**Theoretical Biomass Potential (t ha^-1^)**			**Theoretical Energy Potential (GJ ha^-1^)**			**Available Energy Potential (GJ ha-1)**		
				Max.	Min.	Avg.	Max.	Min.	Avg.	Max.	Min.	Avg.
*Brassica carinata*	2017–2018	Straw	10.93 ± 0.19	10.92	6.03	7.92 ± 0.16	129.122	70.76	93.61 ± 1.84	101.91	48.39	68.06 ± 1.58
	2018–2019	Straw	10.44 ± 0.19	10.71	5.82	7.61 ± 0.16	124.08	67.51	88.94 ± 1.83	98.32	46.38	65.19 ± 1.59
*Carthamus tinctorius*	2017–2018	Straw	2.76 ± 0.12	1.92	0.42	1.19 ± 0.06	28.75	4.65	14.98 ± 0.81	13.76	1.57	6.53 ± 0.42
	2018–2019	Straw	2.61 ± 0.11	1.79	0.41	1.13 ± 0.06	24.17	4.39	14.22 ± 0.80	11.92	1.51	6.22 ± 0.42

a Oilseed crops production quantities produced annually.

TBP was found to be very different for each of the two crops. In the case of Ethiopian mustard, it ranged from 10.82 to 5.93 t ha^−1^ on average over the two-year tests. Safflower, however, had an average value of 1.16 t ha^−1^, with average maximum and minimum values of 1.86 and 0.42 t ha^−1^. TEP was determined on average as 91.27 GJ ha^−1^ (Ethiopian mustard) and 14.60 GJ ha^−1^ (safflower), varying over a wide range in both crops and both growing seasons. AEP was found, on average, to be 66.63 GJ ha^−1^ (Ethiopian mustard) and 6.37 GJ ha^−1^ (safflower). Focusing on Ethiopian mustard, AEP ranged from 100.11 to 47.38 GJ ha^−1^, on average, in the two-year study period; in contrast to safflower, which produced values lying between 12.84 and 1.54 GJ ha^−1^. In general, the results of the assessment show that Ethiopian mustard performed better in terms of energy potential compared to safflower in the area of the study.

### 3.5 Economic profitably of crop cultivation

The results of profit analysis of Ethiopian mustard and safflower cultivation are shown in Table 11.

**Table 11:** Estimation of variable costs of crop production, production value, and gross income for Ethiopian mustard and safflower.

**Items (€ ha^−1^)**	**Ethiopian mustard**	**Safflower**
Sowing	140.00	130.00
Tillage	240.00	240.00
Fertilisation	180.00	150.00
Weeds and pests control	250.00	200.00
Seed harvesting	100.00	100.00
Crop residue harvesting	70.00	70.00
*Variable costs of crop production*	980.00	890.00
Seed sale	754.00	1324.53
Agriculture residue sale	351.00	49.50
*Production value*	1105.00	1374.03
*Gross income*	125.00	484.03

In accordance with the proposed method, the variable costs of crop production were estimated as 980 € ha^−1^ for Ethiopian mustard, while those of safflower resulted 890 € ha^−1^.

The production value was calculated as 1.105 € ha^−1^ for Ethiopian mustard and 1.374 € ha^−1^ for safflower, on average. This difference was mainly due to the highest sale price of safflower seed. In fact, the seed sale price for safflower amounted to 871 € t^−1^ while it was estimated as 260 € t^−1^ for Ethiopian mustard. When considering the agricultural residue sale price, it was estimated as 45 € t^−1^ for both crops. However, it marginally influenced the production value of the two crops despite the highest agricultural residue yield for Ethiopian mustard (7.8 t ha^−1^, as two-year average) with respect to safflower (1.1 t ha^−1^ as two-year average). On this basis, the gross income of safflower (484 € ha^−1^) was assessed to be higher than that of Ethiopian mustard (125 € ha^−1^), highlighting greater economic profitability.

## 4 Discussion

Nowadays, agricultural residues represent a promising source for energy production, enabling a reduction in the negative enviromental impact of the conventional energy sector [11]. Residues are a source of bioenergy and contribute to satisfy the energy demand from farmers [60]. Agricultural residues, once considered as waste, are increasingly being viewed as an important resource with high economic and energetic value, whilst also promoting models of sustainable agriculture [61,62]. In the European Union, energy policies concerning the exploitation of these residues have recently been implemented in order to achieve bioenergy objectives and initiate the transition towards a bioeconomy, as reported by Gérard and Jayet [63]. Some studies highlight the fact that, in numerous countries, farmers value agricultural residues on the same as, or on an even higher level, than grain due to the strategic role they play in the renewable energy market [64,65]. In the Mediterranean region, for example, Zanetti et al. [29] state that farmers who mostly rely on winter cereals are looking for uncommon winter crops with the aim to diversify the cropping systems and respect the agricultural sustainability. Various authors agree with the fact that the introduction of oilseed crops into rainfed cereal-based cropping systems allows farmers to increase agricultural production in terms of both grain and ARYs [31,47,66]. Consequently, oilseed crops may act as a strategic tool, helping to provide raw materials and meet the targets of the European energy policy. Furthermore, oilseed crops would also aid the progress of the agricultural sector, which is often in crisis due to difficulty in finding market-viable alternative products.

With this in mind, two annual oilseed crops were assessed in the present study over two growing seasons in order to determine their yields and the energy potential of the agricultural residues. The results showed that both oilseed crops suitably adapted to the environmental characteristics of the study area, confirming their potential cultivation in the Mediterranean region. They obtained highly relevant differences concerning the morphological parameters mainly due to different origin of the Ethiopian mustard and safflower accessions. The two crops also provided good agronomic performance in terms of seed and ARYs. The yields were significantly influenced by year, genotype and year-by-genotype interactions. Our findings were compared with those obtained in similar environmental conditions and a number of differences were detected.

Regarding Ethiopian mustard, in a study conducted in three Sicilian sites, Copani et al. [67] calculated average seed yield (1.90 t ha^−1^) and residual biomass yield (3.80 t ha^−1^) whose results were lower than those of the present study. In South Italy, in three different sites in the Apulia region, Montemurro et al. [27] made a comparison between different agricultural practices used to realize optimum yield performances for biodiesel generation, obtaining lower productive performances in terms of seed and ARYs. In Central Italy, Del Gatto et al. [26] assessed the agronomic performance of various varieties of Ethiopian mustard and found similar results to our study for seed yield only (2.89 t ha^−1^), whilst those of ARYs (14.30 t ha^−1^) were twofold that of our findings. In Northern Italy, Zanetti et al. [68] investigated the yield trend of some varieties under low and high production inputs and recorded an average seed yield of 2.90 t ha^−1^. In the case of safflower, Abou Chehade et al. [30] conducted 2-year field study in Italy to assess how genotype and growing season influenced the yield performance of the crop and found a higher average seed yield (1.93 t ha^−1^) compared to our findings. Zanetti et al. [29] assessed the suitability of high-oleic safflower, taking into consideration multiple growing seasons andeight locations in Emilia Romagna and Tuscany (Italy), obtaining very different results for ARY (6.49 t ha^−1^ on average). In Sicily, La Bella et al. [28] assessed the yield performance of several safflower accessions to verify their adaptation capacity to specific climate conditions and found lower results for seed yield (1.11 t ha^−1^). Worldwide, various authors [[69], [70], [71]] found different findings both for seed and ARYs when assessing the effects of diverse agronomic practices on safflower yield performance. For both crops, the previously cited studies highlight that differences in yield parameters depend on cultivation practices, environmental conditions, and genotype traits, mainly. For example, the negative impact of hot and dry climate conditions on SY and ARY of safflower is confirmed by a number of studies [29,71,72]. Other studies report contrastating results regarding the impact of various agronomic practices on Ethiopian mustard yield [[73], [74], [75]]. In our study, when comparing the two crops, Ethiopian mustard produced higher seed and ARYs, confirming the results provided by literature. In particular, both crops obtained highest yield results during the first growing season. This means that the different air temperatures and rainfall levels between the two years affected plant growth, leading to increases or decreases in SY and ARYs.

It is well known that residues from the two oilseed crops have an agronomic use when buried. Abou Chehade et al. [30] affirm that Ethiopian mustard residues remove significant amounts of N and P, which can then be returned to the soil after being incorporated through tillage, leading to improvements in soil fertility, which can then be exploited by crops during rotation. Licata et al. [31] add that difficulty removing safflower root residues from the soil would suggest leaving them in place, thus contributing tothe production of stable humus, respecting the sustainable agriculture criteria. In the energy sector, it has been demonstrated that agricultural residues also have a potential for energy production, especially in rural areas of developing countries, potentially increasing the farmers income [33,76]. However, knowledge of the energetic characteristics of the biomass residues, together with the theoretical and available biomass energy potential, would seem to be essential when selecting oilseed crops for use in agricultural lands and for the production of bioenergy directly on-farm.

Literature provides limited information concerning the assessment of the energetic characteristics of agricultural residues obtained by oilseed crops. In a study conducted to assess the quality of different raw materials provided by some industrial crops, such as cardoon (*Cynara cardunculus* L.), Ethiopian mustard, rapeseed and sunflower, Duca et al. [38] obtained interesting findings on their energy content and energetic potential. When comparing our data with those provided by these authors, great differences were found. In the case of Ethiopian mustard residues, average ash content was similar, whilst average HHV and LHV were much lower than the reference values. As safflower had not been evaluated by the above-mentioned authors, data on sunflower residues were used as reference instead, as this crop belongs to the same botanical family as safflower. Sunflower stalks and heads produced higher average ash content, HHV and LHV with respect to safflower stover. These results allow us to make a number of considerations on the exploitation of these residues in the combustion process for thermal and electrical energy cogeneration, in particular. The energy potential contained in the agricultural residues of the two oilseed crops (based on HHV and LHV), can be regarded as substantial, considering results reported in scientific literature. However, the high average moisture content of these residues negatively affects combustion performance. This means that it is necessary to use strategies to reduce residue moisture content at the harvest site or apply pretreatments when attempting to exploit the energy use of stover [77,78]. Moreover, the high ash content of the oilseed residues could cause management problems during the combustion process and become an element of concern, depending on the size of the combustion plant, as reported by Duca et al. [38].

In our study, the agricultural residues were also tested in pellet-making and the end-product was analysed in energetic and physical terms. The use of Ethiopian mustard and safflower pellets for energy purposes is less documented in literature [79]. For example, Ethiopian mustard pellets created from defatted seed meal is mainly exploited in biofumigation practices to control root pathogens [80]. In a survey conducted in Italy, Duca et al. [81] assessed only the quality of wood pellets, taking into consideration 130 pellet bags made from forest residues. The authors calculated some parameters such us the ash content (0.9 %), moisture content (6.70 %), net calorific value (17.0 MJ kg^−1^) and mechanical durability (98.0 %) of the wood pellets. The wood pellets were superior in terms of physical characteristics and energetic potential with respect to Ethiopian mustard and safflower pellets. This evidence highlights that pellets made from oilseed crops residues do not show the same qualitative characteristics of wood pellets and are modestly suited for energy use. Despite this, these residues could be blended with other agricultural/forest residues on farm to develop circular economy strategies, as stated by Hagos et al. [33].

An important aspect regarding agricultural biomass residues is the quantities in which they are potentially available. In general, this depends on crop production and, thus, the greater the crop production, the greater the residue yields. The knowledge of the potential biomass availability in an area is fundamental to promote renewable energy actions and provide planning based on biomass residue potential, as affirmed by Avcioğlu et al. [22]. At farm level, an estimation of potential biomass availability would be helpful when making decisions on the energy use of the biomass. Potential availability would also provide a simple framework when developing a support tool for farmers, as reported by Andrieu and Nogueira [82]. Various studies were conducted in different countries to evaluate the energy potential of these residues [11,22,51]. All studies took into account agricultural biomass residues in large geographical regions in order to highlight the diversity of agricultural crop types in terms of potential available biomass quantities and biomass potential energy. Most of them underlined the fact that agricultural biomass can potentially be used for energy purposes. Our study was carried out in an experimental area to calculate the energy potential of the available agricultural biomass provided by two oilseed crops, viewed as a renewable resource to exploit together with pure vegetable oil directly on-farm. As a consequence, it was not possible to make comparisons in terms of production data with previous studies due to different area of investigation. Ethiopian mustard performed much better than safflower in terms of theoretical biomass potential based on stover yield in both growing seasons, confirming its excellence as a biomass oilseed crop. It is well-known that various factors can greatly degradate the biomass at storage and transport levels, as reported by Anerud et al. [83]. In our study, these losses were not considered due to the low amount of biomass stored and the fact we carried out trials at experimental scale.

When analysing the indices for measuring the biomass energy potential, it is worth noting that only the exploitation of Ethiopian mustard residues would seem to make sense in the study area, in terms of biomass energetic use, due to higher theoretical energy potential and available biomass energy potential. This means that the two oilseed crops deeply differ from each other not only in terms of biomass availability but also in terms of biomass potential energy, affecting farm decisions on agricultural residue management. In this study, the proposed model of economic analysis permitted to estimate the profitability of the biomass production both for on-farm use and for sale. Based on the main results, the gross income of safflower cultivation was estimated to be higher than that of Ethiopian mustard because of the highest seed sale price. The analysis showed that the crop residue sale price did not affect the production value of the two crops in the same way as the price of seed. Although the lowest net profit margin of Ethiopian mustard cultivation, the findings of the present study suggest that Ethiopian mustard biomass, in particular, represents a promising and prospective source of energy production directly on-farm but at the same time highlight some problems which need first to be solved. Firstly, the continuity and sustainability of biomass residues are fundamental for the comparison and selection of oilseed crops and the establishment of biomass power plants, as confirmed by Avcioğlu et al. [22]. Secondly, the energetic exploitation of agricultural residues has to ensure continuity and stability in food production at farm level, in accordance with Gavrilescu [16].

## 5 Conclusions

In rural areas of the Mediterranean region, Ethiopian mustard and safflower are two dedicated annual oilseed crops that provide different productive performances under rainfed conditions. At farm level, the valorisation of agricultural biomass residues, as a valuable feedstock to pure vegetable oil for producing renewable energy, can represent a model of circular economy and contribute to satisfy the sustainable agriculture criteria. These residues produce bioenergy during various processes, however knowledge on their energetic characteristics and calculation of the theoretical and available biomass energy potential appear fundamental for planning energetic production based on biomass directly on-farm. In the present study, the energy potential of agricultural residues, based on higher and lower heating values, was estimated as satisfactory for both oilseed crops; however, moisture and ash contents were found too high, requiring specific pretreatments. Pellets made from agricultural residues were found to be modestly suited to energy use. Ethiopian mustard performed far better than safflower in terms of theoretical energy potential and available biomass energy potential and could be considered of greater interest for energy purposes. Due to fact that this survey was conducted on an experimental scale, it is not possible to affirm that the estimated biomass quantification potentially ensures a sustainable source of energy that could help meet the energetic needs of a farm. Although agricultural residues can be deemed as a method to increase income for farmers, various factors must be taken into consideration in the future regarding the availability and continuity of agricultural residues of both crops, many of which depend on energy policies and government incentives for oilseed crops. Furthermore, other factors, such as agricultural lands available, oilseed crop allocation, competition with food production and climate change, must be considered to successfully exploit agricultural residues as renewable forms of energy on farm.

## Funding statement

This study received grant from the 10.13039/501100003407Italian Ministry of Education, University and Research (grant numbers CTN01_00063_49295).

## Additional information

No additional information is available for this paper.

## CRediT authorship contribution statement

**Mario Licata:** Writing – review & editing, Writing – original draft, Visualization, Validation, Methodology, Investigation, Conceptualization. **Davide Farruggia:** Writing – review & editing, Writing – original draft, Software, Formal analysis, Data curation. **Filippo Sgroi:** Writing – review & editing, Writing – original draft, Validation, Methodology, Investigation, Data curation. **Francesco Salamone:** Software, Data curation. **Claudio Leto:** Resources, Project administration, Funding acquisition. **Giuseppe Di Miceli:** Writing – review & editing, Visualization, Supervision, Resources, Project administration.

## Declaration of competing interest

The authors declare that they have no known competing financial interests or personal relationships that could have appeared to influence the work reported in this paper.
